# Can emotional content be extracted under interocular suppression?

**DOI:** 10.1371/journal.pone.0206799

**Published:** 2018-11-12

**Authors:** Yung-Hao Yang, Su-Ling Yeh

**Affiliations:** 1 Department of Psychology, National Taiwan University, Taipei, Taiwan; 2 Graduate Institute of Brain and Mind Sciences, National Taiwan University, Taipei, Taiwan; 3 Neurobiology and Cognitive Science Center, National Taiwan University, Taipei, Taiwan; 4 Center for Artificial Intelligence and Advanced Robotics, National Taiwan University, Taipei, Taiwan; University of Florida, UNITED STATES

## Abstract

Previous studies showed that emotional faces break through interocular suppression more easily compared to neutral faces under the continuous flash suppression (CFS) paradigm. However, there is controversy over whether emotional content or low-level properties contributed to the results. In this study, we directly manipulated the meaningfulness of facial expression to test the role of emotional content in breaking CFS (b-CFS). In addition, an explicit emotion judgment for different facial expressions (happy, neutral, and fearful) used in the b-CFS task was also conducted afterwards to examine the relationship between b-CFS time and emotion judgment. In Experiment 1, face orientation and luminance polarity were manipulated to generate upright-positive and inverted-negative faces. In Experiment 2, Asian and Caucasian faces were presented to Taiwanese participants so that these stimuli served as own-race and other-race faces, respectively. We found robust face familiarity effects in both experiments within the same experimental framework: upright-positive and own-race faces had shorter b-CFS times than inverted-negative and other-race faces, respectively. This indicates potential involvement of high-level processing under interocular suppression. In Experiment 1, different b-CFS times were found between emotional and neutral faces in both upright-positive and inverted-negative conditions. Furthermore, with sufficient duration (1000 ms) participants could still extract emotional content in explicit valence judgment even from inverted-negative faces, though with a smaller degree than upright-positive faces. In Experiment 2, differential b-CFS times were found between emotional and neutral faces with own-race but not other-race faces. Correlation analyses from both experiments showed that the magnitude of emotion judgment was correlated with b-CFS time only for familiar (upright-positive / own-race) but not unfamiliar (inverted-negative / other-race) faces. These results suggest that emotional content can be extracted under interocular suppression with familiar faces, and low-level properties in unfamiliar faces may play a major role in the b-CFS time.

## Introduction

Understanding emotional content of facial expression is a critical skill for social interaction and communication. Since one must be sensitive to the emotional context to engage in an effective communication, it is important to detect emotional expression rapidly, automatically, or sometimes even unconsciously. However, whether emotional face, rather than neutral face, can gain prioritized access under unconscious processing still remains a hotly debated and yet unsolved issue. In this study, we adopted the breaking continuous flash suppression (b-CFS) [1–2] paradigm to examine whether emotional content can influence breakthrough times under CFS when different facial expressions were presented and masked. When using the b-CFS technique, a series of dynamic high-contrast masks are presented to one eye, and due to the interocular suppression, this renders a critical stimulus presented to the other eye invisible. By manipulating the contrasts of the critical stimulus on one eye and the flashing mask on the other eye, eventually the interocular suppression from the mask would break and the critical stimulus would become visible. The reaction time required for b-CFS is usually considered as an index of how efficiently a stimulus breaks through interocular suppression and becomes visible [3].

By adopting this approach, several studies have demonstrated that facial expression can be processed under CFS [4–5], but it is currently still being debated whether the results should be attributed to emotional content or low-level properties such as local contrast, spatial frequency, etc. [6–7]. For example, E. Yang et al. [5] found that fearful faces broke interocular suppression more quickly than neutral and happy faces, and similar results were also observed when faces were inverted or even when only the eye regions of different facial expressions were presented. The authors thus concluded that salient eye-white regions embedded in fearful faces contributed to the preferential access of fearful to neutral/happy faces. Nevertheless, while the salient eye-white region with high local contrast could be necessary to represent a fearful face, the high local contrast should not be equated to fearful face since most people would not label or feel that high contrast is fearful. Critically, the information from the eye-white regions cannot be used by the amygdala lesioned patient SM to recognize fearful faces [8], but later the study still revealed rapid detection of the fearful face using the b-CFS paradigm for this patient [4]. Taken together, these findings raised a question: is the finding of preferential access of facial expression under b-CFS simply due to sensory efficiency (e.g., high local contrast of the eye white region in fearful face) without accessing the emotion meaning of the face, or does emotional content (e.g., potential treats) also play a role in the differential b-CFS time?

To examine whether emotional content can be extracted under interocular suppression, it is imperative to incorporate a control condition that modulates meaningfulness of emotional content but can nonetheless keep low-level properties similar across conditions [3]. One common practice is to use upright and inverted faces for controlling low-level properties. The basic idea is that if emotional content is a critical factor and is successfully interrupted in the control condition, the difference in b-CFS time among different emotions in this control condition should not be observed. On the other hand, if the b-CFS time difference is still observed in the control condition, one would conclude that emotional content is not critical to determine the b-CFS time. Rather, it is inferred that low-level information determines the b-CFS time.

Nevertheless, manipulating face orientation (upright vs. inverted) alone seems insufficient as an unambiguous control to dissociate low-level from high-level information in facial expression, as shown in previous studies (e.g., [5]). In fact, the effectiveness of impairment from face inversion is still debated: while some suggest recognitions of all kinds of emotional expressions are impaired by inverting the faces [9], others report that emotional expressions are less affected by face inversion [10–12]. Therefore, even though similar b-CFS times between upright and inverted faces were found, the findings could not be used to clarify unequivocally whether the results were due to emotional content, or were contributed solely by low-level properties [6].

To address this issue, Gray et al. [6] combined face inversion with negative luminance polarity to generate a control face (termed *inverted-negative face* hereafter) condition, which interrupted emotional information more severely than inversion alone but kept the low-level properties similar to the original face condition (upright and positive polarity of luminance; termed *upright-positive face* hereafter). Their results showed that emotion discrimination of inverted-negative face was at chance level in an explicit emotion judgment task when the stimuli were presented briefly (100ms), indicating that there was no processing of emotional content for the inverted-negative face. More importantly, the inverted-negative face revealed similar pattern to the upright-positive face condition in the b-CFS task; that is, fearful faces led to shorter breakthrough times compared to neutral and happy faces in both inverted-negative and upright-positive conditions. Therefore, the authors concluded that the preferential access for fearful faces under interocular suppression could be entirely explained by the *low-level properties* since there was no processing of emotional content as revealed by the emotional judgment task.

Gray and Colleague’s conclusion was based on the assumption that inverted-negative face is an ideal control for “no emotional content” [6]. However, this assumption may not be valid for the following reasons. First, the inverted-negative faces in their explicit emotional judgment task were only presented for 100 ms, but the b-CFS time usually took several seconds (e.g., 3~4 sec in [6]) to process. Therefore, the face presentation in the explicit emotional judgment task could have been too short to reveal emotional content (if any). Second, in addition to a similar pattern of b-CFS times across different facial expressions between upright-positive and inverted-negative conditions, the overall b-CFS times were shorter in upright-positive faces than inverted-negative faces. Even though this result could be explained by familiarity of orientation and luminance polarity, a certain level of emotional content might have contributed to the different overall b-CFS times across manipulations as well, since it also gets increasingly difficult (as shown by their explicit emotional judgment task) to extract emotional content as one manipulates the orientation and the polarity of the face stimuli. Hence, while low-level features may seem sufficient to explain the shorter b-CFS times of fearful facial expression, the possibility of emotional processing under interocular suppression is still not completely ruled out.

In the present study, we tackled this issue with two sets of b-CFS experiments that manipulated the meaningfulness of emotional content. We also adopted a modified emotion judgment task by presenting the faces for 1000 ms to allow for the participants sufficient processing time. While the processing of this explicit judgment is not necessarily equal to the effectiveness of the facial expression under interocular suppression, this task can serve as a manipulation check to reflect participants’ subjective feeling of emotional content the face contains. If the processing of facial expression under interocular suppression is driven by emotional valence, the b-CFS time difference should reflect the difference in emotional content.

In the first experiment, we used inverted faces with negative luminance polarity to compare with upright-positive faces as in Gray et al. [6]. Nevertheless, longer presentation time of faces in the explicit emotion judgment task would provide sufficient processing time to measure if any emotional content is still preserved in the inverted-negative faces condition. In the second experiment, we incorporated yet another familiarity factor, race familiarity, as a factor (own-race faces vs. other-race faces) to modulate emotional content of facial expression, as own-race familiarity has been shown to influence emotional recognition of facial expression [13–14]. That is, participants performed better when recognizing emotion for faces from the same race as them than for faces from other races [15]. To examine whether breakthrough times of facial expression under interocular suppression is driven by meaningfulness of emotional content, additional correlation analyses between b-CFS time and emotion judgment in both experiments were conducted.

## Experiment 1

The purpose of the current experiment was to understand the relationship between the role of emotional content and processing efficiency of facial expressions under interocular suppression, by manipulating one of the two familiarity factors (face orientation and contrast polarity) in this study. In this experiment, we adopted inverted-negative face stimuli to compare with upright-positive face stimuli as utilized by Gray et al. [6]. We also asked the participants to rate the emotional valence for each facial stimulus after the main b-CFS task. Correlation analysis between the suppression time in the b-CFS task and the emotion judgement made by each participant was conducted.

The explicit emotion judgment task was a manipulation check to support how much emotional meaning was still preserved in these faces. If emotional content was completely abolished in inverted-negative faces as suggested by Gray et al. [6] our prolonged presentation should reveal that participants cannot judge the emotional valence of the faces effectively. Following this, if the processing efficiency of facial expressions under CFS is driven by emotional content of the faces, then the suppression times should reveal a different pattern of the three facial expressions between upright-positive faces and inverted-negative faces. The b-CFS times should also fluctuate with the meaningfulness of emotional content and significant correlation between suppression time and emotion judgment should be observed.

### Method

#### Participants

We determined the sample size based on the standardized effect size (dz = .61) from a critical comparison of E. Yang et al. [5], where the difference between upright-happy (M = 3.59 s, SD = .9 s) and upright-fearful (M = 2.98 s, SD = 1.01 s) faces was significant in a face detection task (Experiment 1) under CFS. To achieve a power of (1 − β) = .8 with Type I error of α = .05, the G*Power 3.1 toolbox [16] suggested a sample size of 22.

Twenty Taiwanese participants were recruited in this experiment. They were naïve about the purpose of this experiment and had normal or corrected-to-normal vision. Ethical approval for this study was granted by the Ethics Committee of the Department of Psychology, National Taiwan University. All of the participants gave written informed consent according to the rules of the Ethics Committee of the Department of Psychology, National Taiwan University.

#### Stimulus materials and apparatus

Visual stimuli were presented on a 21-inch Eizo T966 CRT monitor (1024 × 768 resolution at 60 Hz refresh rate) and controlled by Matlab 2012a (the Math Works, Natick, USA) with Psychophysics Toolbox 3.0 [17–18] on a Microsoft Windows PC. Participants viewed stimuli through a four-mirror stereoscope, such that two fusion contours (10.6° × 10.6°, 0.3° thickness with white noise pattern) with gray background (RGB values of 127, 127, 127) could be respectively presented to different eyes to maintain stable fusion. A white fixation-cross (0.7° × 0.7°; RGB values of 255, 255, 255) was displayed in the center of each fusion contour, and participants were asked to focus on the fixation throughout each trial. A face stimulus (4.5° × 4.5°) was presented on one of the four quadrants (upper/lower and left/right) in one fusion contour (center-to-center distance: 3.3°), and the Mondrian-like masks that contained 1000 small patches (random size: 0.02° to 1.0°; random gray scale: 0~255) were presented in the other fusion contours. To decrease response variance between dominant eye and non-dominant eye within an observer, the eye presented with the suppressed faces were kept constant within each participant. Additionally, the suppressed face that was presented in the dominant eye or non-dominant eye was counterbalanced between participants.

**Fig 1:**
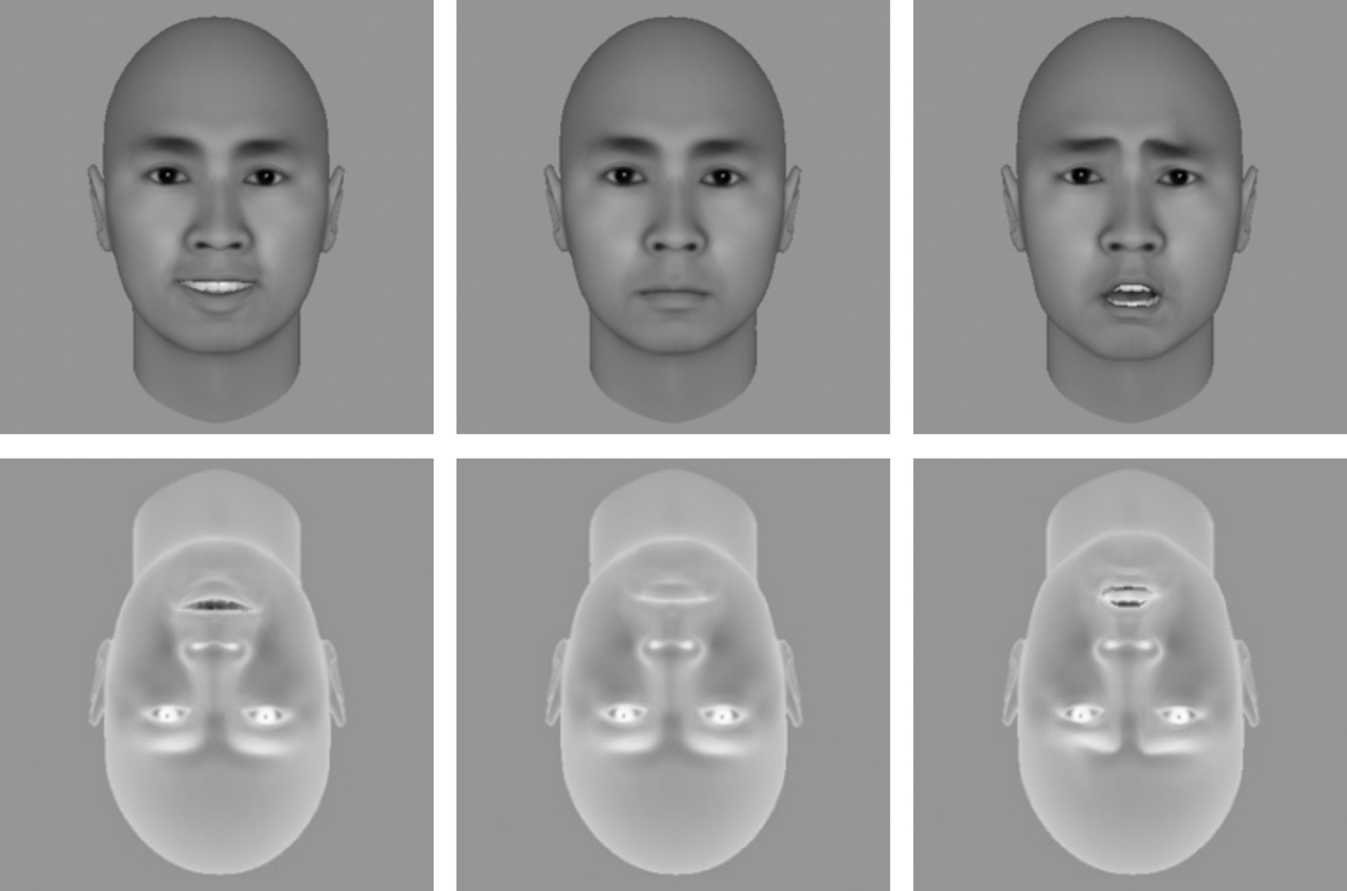
Samples of stimuli used in Experiment 1. Upright-positive faces are shown in the top panels and inverted-negative faces in the bottom panels. Happy, neutral, and fearful faces are shown in the left, middle, and right panels, respectively.

Eight posers (4 females) were generated by the FaceGen software (Singular Inversions Inc.), each with three facial expressions (happy, neutral, and fearful). The mean luminance and root mean squared (RMS) contrast of the face stimuli were matched across different facial expressions. These facial stimuli were either presented in upright orientation with positive luminance polarity (upright-positive face), or in inverted orientation with negative luminance polarity (inverted-negative face; Fig 1). To avoid abrupt onset triggering a dominant face percept at the beginning of each trial, the contrast of the face was ramped up from 0% to 100% within the first sec after stimulus onset and remained at the highest contrast afterwards [5]. The contrast of Mondrian-like masks stayed at 100% for the first second and was ramped down from 100% to 0% within the remaining 5 seconds.

Following the b-CFS task, an explicit emotion judgment task was given to assess the subjective evaluation for the valence of each face stimuli. The face stimuli (4.5° × 4.5°) was presented in the upper visual field for 1000 ms. After that, participants pressed left- and right-arrow keys to rate the emotional valence from 1 (negative) to 7 (positive) by moving a white square on a rating scale.

#### Design

This experiment contained two tasks, one was the b-CFS task, and the other one was the explicit emotion judgment task. In the b-CFS task, a within-subjects design with face manipulation (upright-positive and inverted-negative) and facial expression (happy, neutral, and fearful) was implemented. Face manipulation, facial expression, face poser, and visual quadrant (where a face would appear on the screen) were orthogonally manipulated and repeated twice, adding up to 384 trials in total. The order of the above combined conditions was pseudorandomized to keep an equal number of trials in each condition. In the explicit emotion judgment task, the 48 faces (8 posers with 3 facial expressions each presented in either upright-positive or inverted-negative settings) were presented in a random order.

#### Procedure

**Fig 2:**
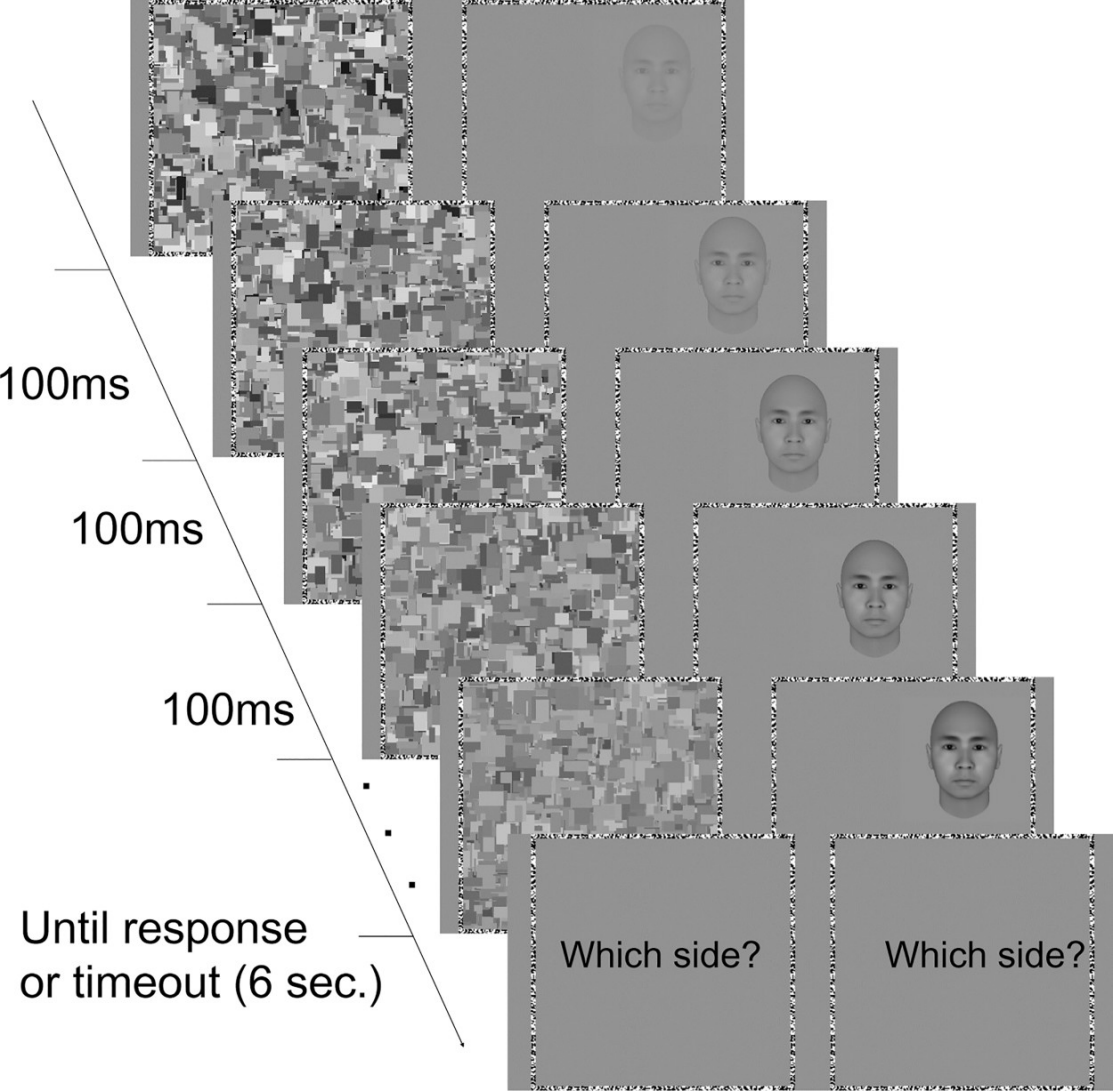
Stimuli and procedure. The face was presented on one of the four quadrants in one eye and the masks were presented for 100 ms per frame in the other eye. The contrast of faces increased gradually from 0% to 100% within the first second and stayed constant until the end of that trial. After 1 second from the onset of the trial, the contrast of masks was ramped down from 100% to 0% for 5 seconds. The trial stopped when participants pressed a key to indicate detection of the face, or after 6 seconds if no response was made. After detection, participants answered which side (left or right) they perceived the face.

Before the b-CFS task, participants viewed the display through the stereoscope and were instructed to adjust the distance between two fusion contours by pressing ‘o’ and ‘p’ keys so the two contours would fuse perfectly. After that, participants pressed the ‘z’ key to start each trial. In each trial, a face was presented in one of the four quadrants to one eye, while Mondrian-like masks flashing at 10 Hz were presented to the other eye. Participants were instructed to press the ‘z’ key again as soon as possible if they detected any part of a face. If they did not press the key for 6 seconds after stimulus onset, the trial would be terminated automatically. After that, they were also required to judge the side of the screen where the face was presented by pressing ‘o’ or ‘p’ key for left and right, respectively (Fig 2).

After the b-CFS task, participants judged the valence of the faces on a 7-point Likert scale in the explicit emotion judgment task. Number 1 referred to negative valence (fearful), number 4 referred to ambiguous valence, and number 7 referred to positive valence (happy) on the scale. In each trial, the face was presented for 1000 ms, and then the participants used the left and the right arrow keys to judge the valence from 1 to 7 without time limit.

### Results

#### Explicit emotion judgment task

**Fig 3:**
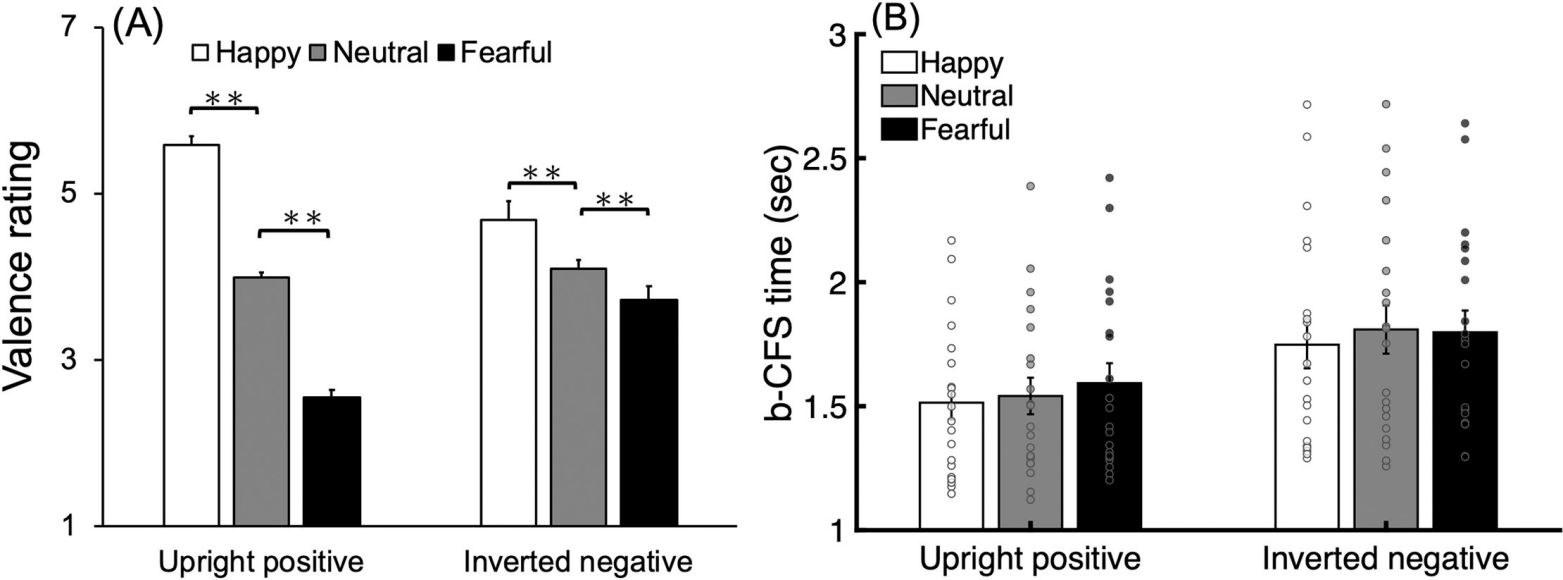
Results in Experiment 1. (A) Valence rating in the explicit emotion judgment task. Scale 1 referred to negative valence and scale 7 referred to positive valence. (B) Mean b-CFS time for detecting upright-positive and inverted-negative faces with happy, neutral, and fearful facial expression. Error bars represent one standard error from the mean and the scatter plot represents the individual data. * p < .05; **p < .01.

The data were analyzed using IBM SPSS Statistics 20.0. A two-way repeated measures ANOVA with factors of face manipulation (upright-positive and inverted-negative) and facial expression (happy, neutral, and fearful) revealed a significant main effect of facial expression [*F*(2,38) = 149.827, *MSE* = .269, *p* < .0001, *η*_*p*_^*2*^ = .887]. Tukey’s HSD post hoc test showed that happy faces (*M* = 5.134, *SD* = .887) were judged more positively than neutral faces (*M* = 4.041, *SD* = .405, *p* < .01), which were judged more positively than fearful faces (*M* = 3.131, *SD* = .847, *p* < .01). There was also a significant interaction between face manipulation and facial expression [*F*(2,38) = 46.699, *p* < .001, *η*_*p*_^*2*^ = .711]. The simple main effect of facial expression in the upright-positive faces [*F*(2, 76) = 4.324, *MSE* = .003, *p* = .0166, *η*_*p*_^*2*^ = .102] revealed that happy faces (*M* = 5.588, *SD* = .420) were judged more positively than neutral faces (*M* = 3.987, *SD* = .308, *p* < .01), which were judged more positively than fearful faces (*M* = 2.550, *SD* = 0.390, *p* < .01). Interestingly, the simple effect of facial expression in the inverted-negative condition revealed a narrower range of emotion judgments, which was nonetheless similar to that of upright-positive faces: happy faces (*M* = 4.681, *SD* = .993) were judged more positively than neutral faces (*M* = 4.094, *SD* = .477, *p* < .01), which were judged more positively than fearful faces (*M* = 3.712, *SD* = 0.779, *p* < .05). There was no main effect of face manipulation [*F*(1,19) = .479, *p* = .497, *η*_*p*_^*2*^ = .025] (Fig 3A).

#### b-CFS task

In the b-CFS task, the b-CFS time from trials with incorrect location judgment were excluded from the analysis (*Mean accuracy* = 98.70%, *SD* = 2.92%). A two-way repeated measures ANOVA with factors of face manipulation (upright-positive and inverted-negative) and facial expression (happy, neutral, and fearful) on mean b-CFS time was conducted (Fig 3B). The main effects of face manipulation [*F*(1,19) = 35.029, *MSE* = .048, *p* < .001, *η*_*p*_^*2*^ = .648] and facial expression [*F*(2,38) = 6.883, *MSE* = .006, *p* = .003, *η*_*p*_^*2*^ = .266] were significant. Upright-positive faces (*M* = 1.549 sec, *SD* = .332 sec) were detected more quickly than inverted-negative faces (*M* = 1.785 sec, *SD* = .419 sec). Tukey’s HSD post hoc test showed that happy faces (*M* = 1.631 sec, *SD* = .387 sec) were detected more quickly than neutral faces (*M* = 1.675 sec, *SD* = .408 sec, *p* < .05) and fearful faces (*M* = 1.695, *SD* = .389, *p* < .01 and no significant difference between neutral and fearful faces (*p* > .05). There was neither significant interaction between face manipulation and facial expression [*F*(2,38) = 1.587, *p* = .218, *η*_*p*_^*2*^ = .077]. We also conducted log-transformed b-CFS time with ANOVA analysis due to the positive skew of raw data. Similar pattern was found for face manipulation [F(1,19) = 50.687, MSE = .002, p < .001, ηp2 = .727], for facial expressions [F(2,38) = 8.78, MSE < .001, p = .001, ηp2 = .316], and for interaction [F(2,38) = 1.245, p = .299, ηp2 = .061]. Tukey’s HSD post hoc test showed that happy faces were detected more quickly than neutral faces (p < .05) and fearful faces (p < .01)

To ensure that the observed effect was not driven by specifically selected faces, we carried out linear mixed effect model (LME) analysis by using the ‘lme4’ package [19] with likelihood-ratio tests from the statistical analysis software R. A full model containing two random intercepts for face identities and for participants, and two fixed effects for face manipulation and facial expression was compared with a reduced model without the fixed effect in question (i.e., face manipulation or facial expression) to test the main effect. The full model plus face manipulation-by-facial expression interaction was compared with the full model to test the interaction effect. Similar results as the ANOVA analysis with raw b-CFS time was found for face manipulation [χ^2^(1) = 28.754, p < .001], for facial expression [χ^2^(2) = 9.46, p = .009], and for interaction [χ^2^(2) = 1.96, p = .376]. The Tukey post hoc test showed a significant difference between happy face and fearful face (p = .007), whereas difference in b-CFS time between happy face and neutral face (p = .142), or neutral face and fearful face (p = .477) was not significant. Similar results were obtained when conducting the analyses on log-transformed b-CFS time, for face manipulation [χ^2^(1) = 30.951, p < .001], for facial expression [χ^2^(2) = 13.22, p = .001], and for the interaction [χ^2^(2) = 1.709, p = .426]. The Tukey post hoc test also showed significant difference between happy face and fearful face (p = .001), whereas b-CFS time for neutral face did not differ from that for happy face (p = .079) or fearful face (p = .308). These LME results suggest that the influence of face manipulation and facial expression under CFS were persevered after accounting for variability across face identities.

#### Correlation analysis

**Fig 4:**
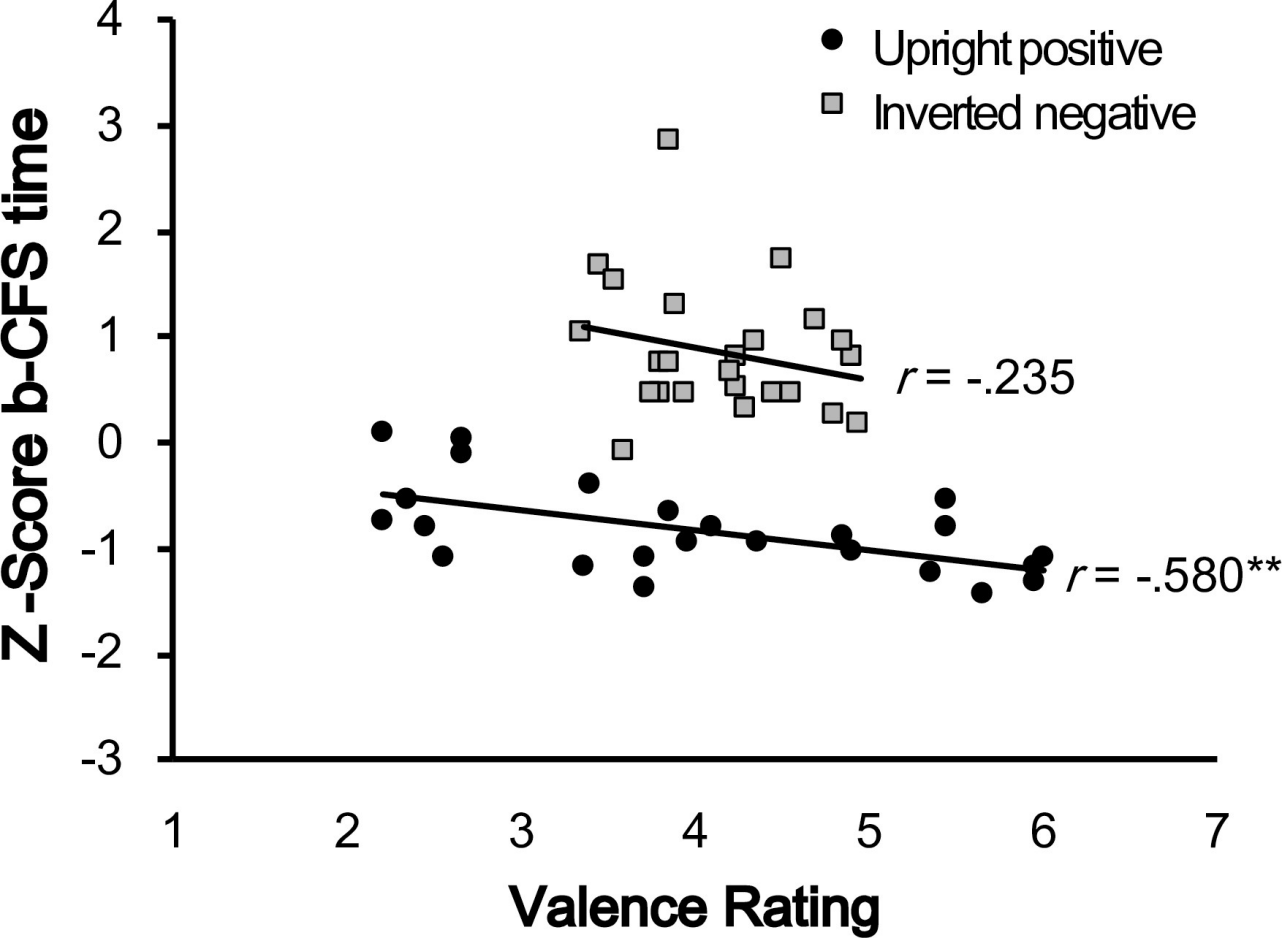
Correlation analysis in Experiment 1. There was significant correlation between b-CFS time and valence rating (1 = negative valence and 7 = positive valence) in the upright-positive faces (black circles) but not in the inverted-negative faces (gray triangles). **p < .01.

To further reveal the relationship between the meaningfulness of emotional content and the breakthrough times of facial expressions under interocular suppression, we also calculated the correlation coefficient between normalized b-CFS time and emotion judgment for upright-positive and inverted-negative faces, respectively. The result showed that the b-CFS time was significantly correlated with the emotion judgment in upright-positive faces (Pearson’s r = -.580, p = .003) but not in the inverted-negative faces (*Pearson’s r* = -.235, *p* = .268; Fig 4).

### Discussion

In this experiment, we adopted inverted-negative faces to compare with upright-positive faces in the current experiment as in Gray et al. [6]. Consistent with previous studies [6, 20], we found that upright-positive faces were detected faster than inverted-negative faces under CFS. This result implies that familiarity factors such as face orientation and contrast polarity of the face stimuli affect processing under interocular suppression. Also, the result showed that the range of emotion judgment for inverted-negative faces was restricted, supporting that the manipulation of inverted-negative face was successful and it had an impact on the processing of facial expression.

However, the assumption that reversing the orientation and contrast polarity was enough to completely disrupt emotional information was not supported. Our data showed that although emotion judgments of inverted-negative faces were shifted toward neutral valence, participants could still differentiate their emotional valences when face stimuli were presented for 1000ms. Thus, we deduced that the inverted-negative manipulation, while impactful, was insufficient to completely disrupt emotional content, and therefore was insufficient to render facial expressions indistinguishable from one another.

As a result, the similar b-CFS magnitude differences between happy and neutral/fearful faces in both upright-positive and inverted-negative conditions we found here can be explained in one of the two ways: emotional content or low-level properties. As emotional content was still preserved in the inverted-negative faces, it would lead to differential b-CFS times between emotional faces and neutral faces. That is, emotional content can be processed under CFS. On the other hand, it could also be possible that low-level properties play a more important role. Note that while we matched the contrast and luminance among different face manipulations, other low-level properties such as phase-spectrum still differed between upright-positive and inverted-negative faces. However, *effective contrast*, the property that Hedger et al. [7] suggested to be a critical factor for the visibility under CFS, was kept the same for both types of stimuli in this experiment. This similar effective contrast thus provides one of the possible mechanisms to explain similar b-CFS patterns for the two conditions. Indeed, later studies using inverted-negative faces as a control condition should bear in mind that emotional information is not completely abolished when given sufficient processing time (e.g., [21]) and this might lead to a difficulty in finding an unequivocal explanation for the issue at hand.

We went one step further by having a closer look at the correlation between suppression time and emotion judgment, and found that different mechanisms may have been involved for the upright-positive and inverted-negative conditions. On one hand, we found that b-CFS time was correlated with emotion judgment in the upright-positive face condition, suggesting that emotional content could determine the breakthrough times of facial expression. On the other hand, while inverted-negative faces reserved some emotional meaning (Fig 3A), suppression times were not correlated with these residual emotional contents (Fig 4). It is thus possible that even with the face stimuli masked, emotional content can be extracted and processed under CFS, but only for familiar faces, not for unfamiliar faces. Nevertheless, it is clear from our results that inverted-negative manipulation as a control condition could not provide an unequivocal baseline to dissociate low-level and high-level information in the processing of facial expression. To better address the possibility that emotional content plays an important role in the processing efficiency of facial expressions under interocular suppression, we introduced own-race familiarity as another manipulation to modulate the meaningfulness of emotional content in the next experiment.

## Experiment 2

It has been demonstrated that familiar identity can influence emotional processing of facial expression. For example, Baudouin, Sansone, and Tiberghien [22] found that recognition of facial expression was more accurate for famous faces and familiar faces than their counterparts. Elfenbein and Ambady [15] also found that accuracy of facial expression judgment favored own-race faces than other-race faces, and the other-race disadvantage in facial expression judgment can be improved by cultural exposure to the other-race group. Based on these findings, we tested whether own-race effect can modulate processing efficiency of different emotional expressions (happy, neutral, and fearful) under interocular suppression.

Previously, a study has demonstrated that own-race effect can be observed under CFS [23]. In the study, the authors measured the magnitude of face inversion effect (FIE, i.e., upright face versus inverted face) in the b-CFS paradigm, and manipulated Caucasian faces (own-race) and black faces (other-race) with Caucasian participants. They found that FIE was only observed in the own-race face condition but not in the other-race face condition. This result suggests that visual awareness of faces can be shaped by the experience of social contact.

**Fig 5:**
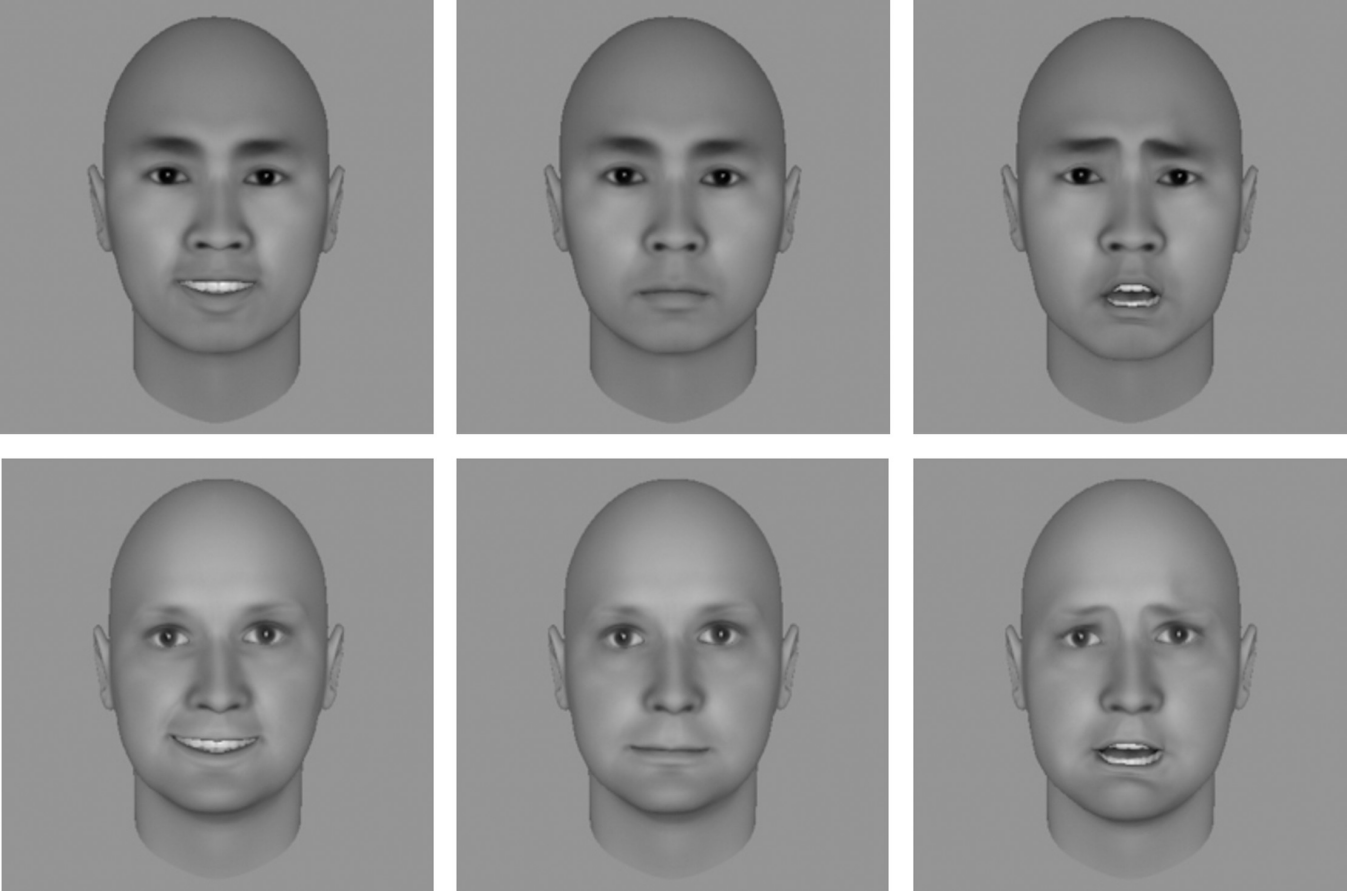
Samples of stimuli used in Experiment 2. Own-race (Asian) faces are shown in the top panels and Other-race (Caucasian) faces in the bottom panels. Happy, neutral, and fearful faces are shown in the left, middle, and right panels, respectively.

To generate the own-race effect, we recruited Taiwanese students as participants and used the same Asian faces as in Experiment 1 (Fig 5, top panel) and Caucasian faces (Fig 5, bottom panel) for the manipulation of own-race and other-race faces, respectively. We made two predictions for this experiment. First, if the previous own-race effect observed with Caucasian participants [23] can be generalized to other races, Asian faces should be detected faster than Caucasian faces for Taiwanese participants. Second, if own-race familiarity can modulate the processing of emotional content in facial expressions, b-CFS time differences among different emotional expressions should be larger in own-race faces than other-race faces.

### Method

#### Participants

Another group of 20 Taiwanese participants were recruited in this experiment. All gave written informed consent and had normal or corrected-to-normal vision.

#### Stimulus, apparatus, design, and procedure

Methods were generally identical to Experiment 1 except for the following. Using the FaceGen software (Singular Inversions Inc.), eight Asian (4 females) and eight Caucasian (4 females) posers were generated to serve as own-race and other-race faces, respectively. Each poser had three facial expressions (happy, neutral, and fearful), resulting in a total of 48 faces. The mean luminance and RMS contrast of the face stimuli were matched across different races and facial expressions. A within-subjects design with the two factors race (own-race and other-race) and facial expression (happy, neutral, and fearful) was implemented in the B-CFS task. In the explicit emotion judgment task, since facial stimuli included both Asian and Caucasian faces, a race judgment task (number 1 referred to Caucasian, number 4 referred to ambiguous race, and number 7 referred to Asian on the scale) was added for each facial stimulus.

### Results

#### Explicit race judgment and emotion judgment task

For the race judgment task, a two-way repeated measures ANOVA with factors race and facial expression revealed a significant main effect of race [*F*(1,19) = 115.931, *MSE* = 2.678, *p* < .0001, *η*_*p*_^*2*^ = .859]; own-race faces (*M* = 5.531, *SD* = .931) were judged more “Asian” than other-race faces (*M* = 2.315, *SD* = .776). There was neither a main effect of valence [*F*(2, 38) = 2.028, *p* = .146, *η*_*p*_^*2*^ = .096 ], nor an interaction between race and facial expression [*F*(2, 38) = 2.260, *p* = .118, *η*_*p*_^*2*^ = .106 ].

**Fig 6:**
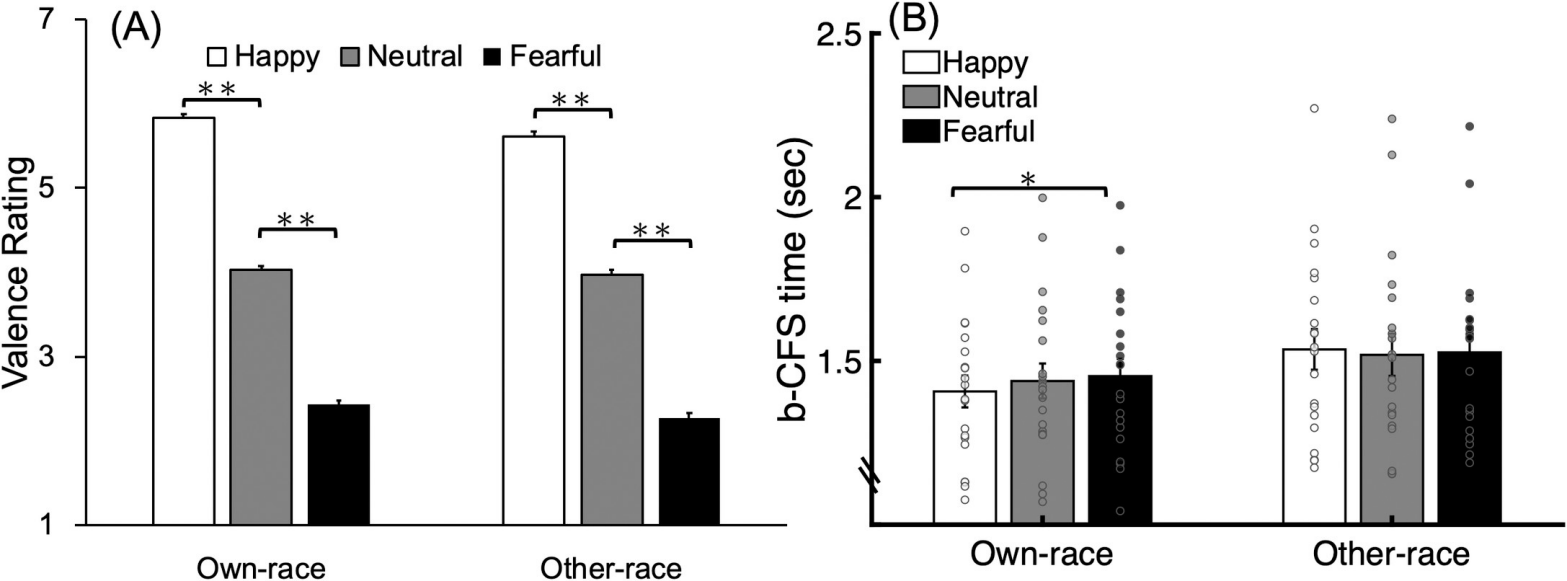
Results in Experiment 2. (A) Valence rating in the explicit emotion judgment task. Scale 1 referred to negative valence and scale 7 referred to positive valence. (B) Mean b-CFS time for detecting own-race and other-race faces with happy, neutral, and fearful facial expression. Error bars represent one standard error from the mean and the scatter plot represents the individual data. * p < .05; **p < .01.

For the emotion judgment task, a two-way repeated measures ANOVA with factors race and facial expression revealed a significant main effect of facial expression [*F*(2,38) = 304.644, *MSE* = .373, *p* < .0001, *η*_*p*_^*2*^ = .941 ]. Tukey’s HSD post hoc test showed that happy faces (*M* = 5.719, *SD* = .481) were judged more positively than neutral faces (*M* = 3.994, *SD* = .370, *p* < .01), which were judged more positively than fearful faces (*M* = 2.35, *SD* = .530, *p* < .01). The main effect of race was also significant [*F*(1,19) = 14.685, *MSE* = .043, *p* = .001, *η*_*p*_^*2*^ = .436 ]; own-race faces (*M* = 4.094, *SD* = 1.465) were judged more positively than other-race faces (*M* = 3.948, *SD* = 1.435). However, there was no interaction between race and facial expression [*F*(2,38) = .721, *p* = .493, *η*_*p*_^*2*^ = .037] (Fig 6A).

#### b-CFS task

The accuracy of location judgment was high (*M* = 99.01%; *SD* = .09%). After removing the data with incorrect judgments of face location, the raw b-CFS times were submitted to further analysis. A two-way repeated measures ANOVA with factors race (own-race and other-race) and facial expression (happy, neutral, and fearful) showed a significant main effect of race [*F*(1,19) = 31.203, *MSE* = .008, *p* < .001, *η*_*p*_^*2*^ = .622 ], reflecting shorter b-CFS time for own-race faces (*M* = 1.433 sec, *SD* = .231 sec) than other-race faces (*M* = 1.527 sec, *SD* = .276 sec), and a significant race by facial expression interaction [*F*(2,38) = 3.731, *MSE* = .002, *p* = .033, *η*_*p*_^*2*^ = .164]. There was no significant main effect of facial expression [*F*(2,38) = 1.278, *p* = .290, *η*_*p*_^*2*^ = .063 ]. Simple main effect of facial expression in own-race faces [*F*(2, 76) = 4.324, *MSE* = .003, *p* = .0166, *η*_*p*_^*2*^ = .102] revealed that happy faces (*M* = 1.407 sec, *SD* = .218 sec) were detected faster than fearful faces (*M* = 1.454 sec, *SD* = .234 sec, p < .05), but no difference between neutral faces (*M* = 1.454 sec, *SD* = 0.234 sec) and happy or fearful faces (ps > .05). There was no significant simple main effect of facial expression in other-race faces [*F*(2, 76) = .511, *p* = .602, *η*_*p*_^*2*^ = .0146 ]. Consistent with the main effect of race, the simple main effect of race in happy [*F*(1,57) = 37.000, *MSE* = .004, *p* < .001], neutral [*F*(1,57) = 14.388, *MSE* = .004, *p* < .001], and fearful [*F*(1,57) = 11.772, *MSE* = .004, *p* < .005] conditions all showed that own-race faces were detected faster than other-race faces (Fig 6B).

We conducted similar analyses as in Experiment 1. In log transformed b-CFS time with ANOVA analysis, similar results were found for race [F(1,19) = 39.275, MSE = .001, p < .001, ηp2 = .674 ], for facial expression [F(2,38) = 1.566, p = .222, ηp2 = .076], and for their interaction [F(2,38) = 3.860, MSE = .001, p = .030, ηp2 = .169]. Simple main effect of facial expression in Asian faces [F(2, 76) = 4.569, MSE < .001, p = .0134] showed that happy faces were detected faster than fearful faces according to Tukey’s HSD post hoc test (p < .05). The analysis of raw b-CFS time with LME analysis yielded significant main effect of race [χ^2^(1) = 9.862, p = .002], but not the main effect of facial expression [χ^2^(2) = 1.853, p = .396]. The race-by-facial expression interaction was marginally significant [χ^2^(2) = 5.488, p = .064]; happy face was different from fearful face in Asian faces (Tukey’s test: p = .0335). There was no other effect (Tukey’s test: ps > .185) The analysis of log-transformed b-CFS time with LME analysis revealed similar result for race [χ^2^(1) = 10.002, p = .002], facial expression [χ^2^(2) = 2.582, p = .275], and interaction [χ^2^(2) = 5.574, p = .062]. Again, the b-CFS time for happy face was different from that for fearful face (Tukey’s test: p = .030) in Asian faces. There was no other effect (Tukey’s test: ps > .183).

#### Correlation analysis

**Fig 7:**
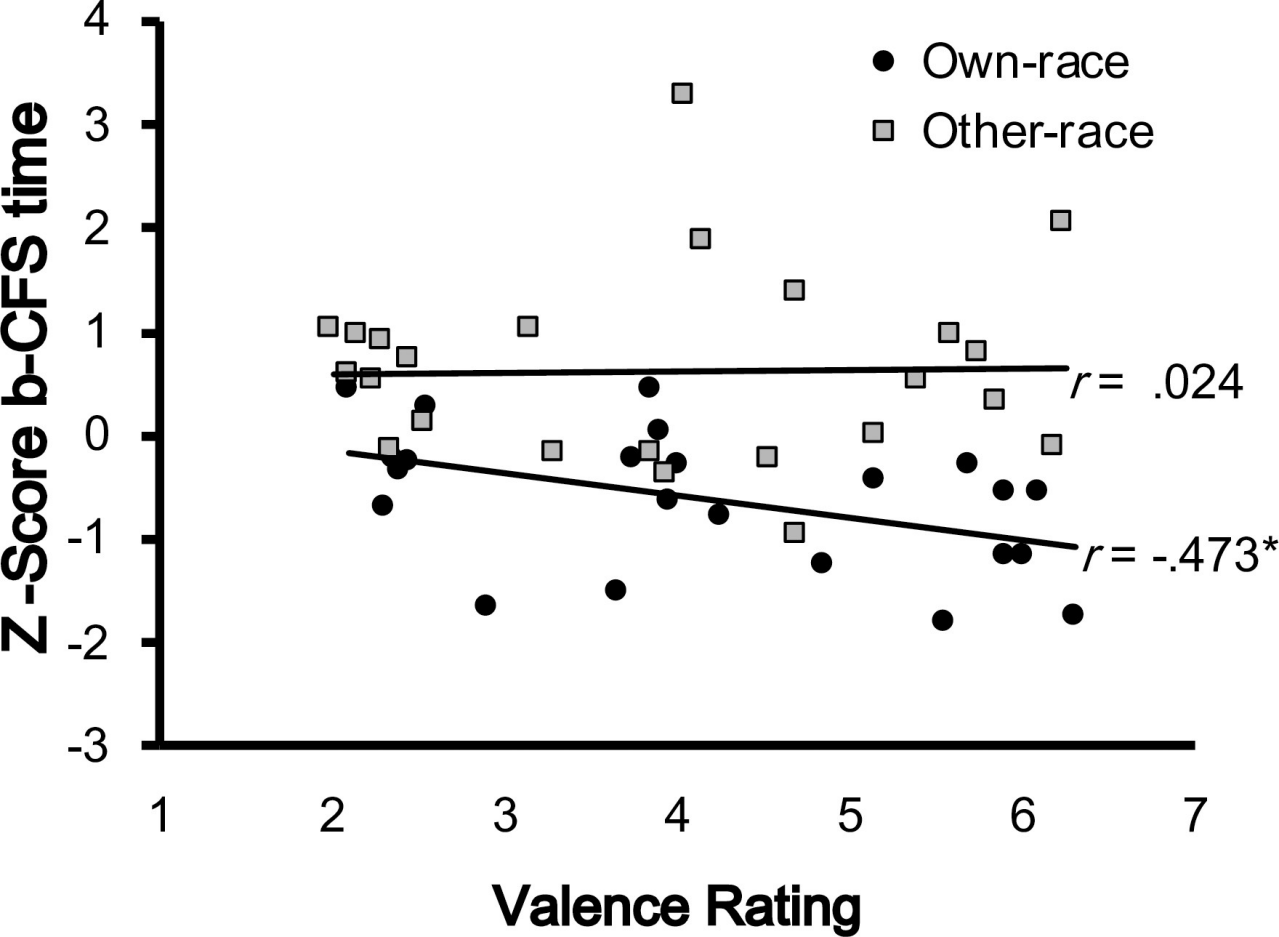
Correlation analysis in Experiment 2. There was significant correlation between b-CFS time and valence rating (1 = negative valence and 7 = positive valence) in the own-race faces (black circles) but not in the other-race faces (gray triangles). *p < .05.

To analyze the suppression time as a function of race and facial expression, we calculated the correlation coefficient between suppression time and valence rating for the two races of face. We used the rating 4 in race rating scale as the cutting point to sort the 48 faces (16 posers, each with 3 facial expressions) into own-race faces and other-race faces. The result showed that participants could clearly distinguish [t(23) = 20.65, SE = .154, p < .001] the Asian faces (M = 5.55, SD = .24) from the Caucasian faces (M = 2.37, SD = .57). Based on this sorting, we found that the normalized b-CFS times significantly correlated with emotion judgment in own-race faces (Pearson’s r = -.473, p = 0.02) but not in other-race faces (Pearson’s r = 0.024, p = .913; Fig 7).

### Discussion

The result of this experiment showed that Taiwanese participants detected own-race faces more quickly than other-race faces. This result was in line with that in Stein et al. [23], providing convergent evidence for the own-race familiarity effect under CFS with the Asian group.

We found that the b-CFS times of emotional expressions were different when viewing own-race faces, so as the significant correlation analysis between suppression times and emotion judgment found in the own-race face. This supports that own-race familiarity could not only be extracted under CFS but also modulate the processing of facial expression. On the other hand, unlike the similar emotion judgment results for both own-race and other-race faces, discrepant patterns of b-CFS time between own-race faces and other-race faces were found. In the other-race condition, emotional contents were preserved but there was no b-CFS times difference among facial expressions. Additionally, no correlation between emotional content and b-CFS times in the other-race faces was found, which would suggest that emotional content alone is not adequate to determine breakthrough times of facial expression for unfamiliar faces like other-race faces.

One possible explanation regarding the different patterns of results for own-race faces and other-race faces could be that the visual system has shaped for frequently seen own-race faces (i.e., Asian face), but not as well for the other-race faces (i.e., Caucasian faces). Consequently, this own-race familiarity influenced the b-CFS time on the processing of facial expression, but this is not the case for the other-race faces.

## General discussion

The purpose of the current study was to test whether emotional content (vs. low-level properties) can be extracted under interocular suppression and can influence breakthrough times of facial expression. To address this issue, we manipulated the meaningfulness of emotional valence on the impact of b-CFS time, and conducted correlation analyses to examine the relationship between emotional content and suppression time under CFS. In Experiment 1, we adopted the inverted-negative faces as stimuli to compare it with upright-positive faces (c.f., [6]). The result revealed that emotional content was still preserved by face inversion and luminance polarity with sufficient processing time, and the b-CFS times among facial expressions were also different for the inverted-negative faces. This result provides empirical evidence questioning the use of inverted-negative faces as a control. In Experiment 2, we adopted other-race faces to compare it with own-race faces and found differential b-CFS times among facial expressions and significant correlation between b-CFS time and emotion judgment only for own-race faces, but not for other-race faces.

Is it possible that some high-level factors still play roles in the b-CFS time? The answer could be “yes” for the following reasons: First, we found consistently face familiarity effect (i.e., face inversion effect and own-race effect) in both experiments, where the upright-positive faces and the own-race faces were detected more quickly than the inverted-negative faces and the other-race faces, respectively. Second, the results showed that the correlation between emotional content and b-CFS times only occurred when viewing familiar faces but not unfamiliar faces in both experiments. These results imply that emotional content could be modulated by the face familiarity effect on the b-CFS time. Third, compared to the face familiarity effect, facial expression only induced a relatively small difference in b-CFS times. This result could be due to the design that unfamiliar faces were mixed with familiar faces, and thus it would dilute the emotional impact on the b-CFS time. In other words, if the result of b-CFS was totally driven by low-level properties, then the b-CFS time difference should not be affected by the proportion of emotional content involved. Taking together, these reasons provide alternative possibilities of high-level modulation to explain our results.

Therefore, the current study suggests that emotional content can be extracted for familiar faces to determine breakthrough times of facial expression, consistent with previous findings for emotional processing with other visual information under b-CFS. Examples of such effect has been documented in previous studies: Schmack, Burk, Haynes, and Sterzer [24] found that breakthrough times of a spider image was modulated by the degree of spider phobia of the observers. Also, Gayet, Paffen, Belopolsky, Theeuwes, and Van der Stigchel [25] showed that neutral stimuli with fear conditioning (e.g., an electric shock) led to shorter b-CFS time than the same stimuli without fear conditioning. Moreover, in our previous study [26], negative described (e.g., fear) or induced (e.g., abuse) words yielded longer b-CFS times than neutral words in the upright word condition, but not in the inverted word or scramble condition. These studies all suggest that emotional content can still influence breakthrough times when low-level properties were relatively similar among different emotional valences conditions.

Compared to the above examples, different facial expressions tested here relied on salient visual characteristics to represent emotional content. Consequently such salient features alone might dramatically influence the breakthrough times of facial expression under interocular suppression [7, 27], like our results of unfamiliar faces here. Therefore, since the b-CFS paradigm is fragile to reflect salient feature attribution that embedded in the facial expressions, other indirect measurements would be more suitable to test the unconscious processing of facial expression. Indeed, recent studies adopted the affective priming with CFS and found that emotional content [28] and own-race familiarity [29] had an impact on subsequent targets. For example, Y.H. Yang and Yeh [28] found facilitated emotion judgment of visible target words by congruent emotional content of invisible face primes under CFS. As this affective priming effect should only be influenced by relevant emotional meaning but not salient features, it thus provides more convincing evidence to support the unconscious processing of facial expression under CFS.

Moreover, the current study replicates previously established effects under CFS and provides additional implications to the literature. In Experiment 1, we adopted inverted-negative faces to compare it with upright-positive faces (c.f., [6]). Previous studies assumed that emotional content of inverted-negative faces can be fully abolished, as reflected by participants’ performance of emotion judgment for these inverted-negative faces. However, when we prolonged the presentation time in the explicit emotion judgment task, the result showed that participants could still discriminate the emotional valence of inverted-negative faces. This result leaves open the possibility that emotional content from inverted (and even luminance polarity reversed) face commonly used as the control baseline in many studies can be preserved under this manipulation [5–6]. This result suggests that a better control condition is needed for studies seeking to test the true contribution of emotional content in facial expression.

In Experiment 2, our results showed that own-race faces were detected faster than other-race faces, consistent with previous findings that familiar faces were processed more efficiently than unfamiliar counterparts under CFS [23, 30]. Compared to a prior study adopting Caucasian faces as own-race face to the Caucasian participants [23], Caucasian faces are the *other-race* to the Taiwanese participants. Therefore, the current finding provides an additional evidence that the own-race effect observed previously is driven by race ownership of participants rather than different low-level properties embedded in faces between different races.

In conclusion, compared to the other studies which focused on the contribution of low-level properties, such as curvature [31], spatial frequency [27], and effective contrast [7], the current study tested the role of emotional content on the breakthrough times of facial expressions. By conducting two experiments manipulating emotional content and examining the correlation between emotion judgment and b-CFS time, we found the reservation of emotional content in the inverted-negative face. This overturns the general assumption that inverted face is a compatible baseline under CFS. Also, we replicated the unconscious own-race effect with Asian group. On top of these observations, we also found that regardless whether the meaningfulness of emotional content was disrupted or reserved, the b-CFS times seem independent of these manipulations for the unfamiliar faces. These results suggest that while emotional content plays some role in the b-CFS times for familiar faces, processing of unfamiliar faces under CFS would be driven by low-level properties.
